# Utilization of Shock Team and Veno-Arterial Extracorporeal Membrane Oxygenation (VA-ECMO) in the Management of Cardiogenic Shock in Northern Ontario

**DOI:** 10.1016/j.cjco.2023.09.019

**Published:** 2023-10-03

**Authors:** Sami Alnasser, Matthew Cavanagh, Rony Atoui, Noman Ali, Bhanu Nalla, Kyle McKechnie, Anthony Main, Mathieu Rheault-Henry, AbdulAziz Al-Shaibi, Lucas Burke, Sarah McIsaac, Robert Anderson, Neil Fam, Mohammed Shurrab, Mary Catherine Kerr, Hooman Hennessey, Craig Armstrong, Bindu Bittira, Abdulrahman Alqahtani, Gregory Papadopoulos, Andreas Kumar, Derek MacDonald, Cormac O’Connor, Michael McDonald, Derek Manchuk

**Affiliations:** a Division of Cardiology, St Michael’s Hospital, University of Toronto, Toronto, Ontario, Canada; b Division of Critical Care, Health Sciences North, Sudbury, Ontario, Canada; c Northern Ontario School of Medicine, Sudbury, Ontario, Canada; d Division of Cardiac Surgery, Sudbury, Ontario, Canada; e Division of Cardiology, Health Sciences North, Sudbury, Ontario, Canada; f Division of Radiology, Health Sciences North, Sudbury, Ontario, Canada; g Division of Cardiology, Peter Munk Cardiac Center, Toronto General Hospital, Toronto, Ontario, Canada

## Abstract

**Background:**

Despite advancements in critical care and coronary revascularization, cardiogenic shock (CS) outcomes remain poor. Implementing a shock team and use of veno-arterial extracorporeal membrane oxygenation (VA-ECMO) have been associated with improved CS outcomes, but its feasibility in remote and rural areas remains unknown.

**Methods:**

This retrospective study included patients with CS who required mechanical circulatory support (MCS) at Health Sciences North, Sudbury, Ontario. The analysis aimed to accomplish 2 objectives: first, to review the outcomes associated with use of Impella (Abiomed, Danvers, MA) and, second, to assess the feasibility of establishing a shock team to facilitate the local implementation of VA-ECMO. The primary endpoint was in-hospital mortality.

**Results:**

The outcomes of 15 patients with CS who received Impella between 2015 and 2021 were reviewed. Their average age was 65 years (standard deviation [SD]: 13), and 8 patients (53%) were female. CS was ischemic in 12 patients (80%). Transfemoral Impella CP (cardiac power) was the most frequently used (93%). Thirteen patients (87%) died during the index hospital stay post-Impella because of progressive circulatory failure. The shock team was established following consultations with several Canadian MCS centres, leading to the development of a protocol to guide use of MCS. There have been 4 cases in which percutaneous VA-ECMO using Cardiohelp (Getinge/Maquet, Wayne, NJ) has been used; 3 (75%) survived beyond the index hospitalization.

**Conclusions:**

This analysis demonstrated the feasibility of implementing a shock team in remote Northern Ontario, enabling the use of VA-ECMO with success in a centre with a sizeable rural catchment area. This initiative helps address the gap in cardiac care outcomes between rural and urban areas in Ontario.

## Résumé

**Introduction:**

En dépit des avancées des soins aux patients en phase critique et de la revascularisation coronarienne, les résultats du choc cardiogénique (CC) semblent mauvais. La mise en place d’une équipe de choc et l’utilisation de l’oxygénation extracorporelle (ECMO, de l’anglais *extracorporeal membrane oxygenation*) par voie veino-artérielle (VA) (VA-ECMO) ont été associées à de meilleurs résultats du CC, mais on ignore sa faisabilité dans les régions éloignées et rurales.

**Méthodes:**

La présente étude rétrospective portait sur des patients en CC qui ont eu besoin d’une assistance circulatoire mécanique (ACM) à Horizon Santé-Nord, à Sudbury, en Ontario. L’analyse visait 2 objectifs : le premier objectif était de passer en revue les résultats associés à l’utilisation de Impella (Abiomed, Danvers, MA) et, le deuxième était d’évaluer la faisabilité de la mise en place d’une équipe de choc pour faciliter la mise en œuvre locale de la VA-ECMO. Le principal critère d’évaluation était la mortalité intrahospitalière.

**Résultats:**

Nous avons passé en revue les résultats cliniques de 15 patients ayant subi un CC qui avaient reçu une Impella entre 2015 et 2021. L’âge moyen était de 65 ans (écart type [ET] : 13), et 8 patients (53 %) étaient des femmes. Le CC était d’origine ischémique chez 12 patients (80 %). L’Impella CP (*cardiac power*, soit la pompe cardiaque) par voie transfémorale était la plus fréquemment utilisée (93 %). Treize patients (87 %) sont morts durant le séjour de référence à l’hôpital après l’utilisation de l’Impella en raison d’insuffisance circulatoire progressive. La mise en place de l’équipe de choc à la suite des consultations dans plusieurs centres canadiens d’ACM a mené à l’élaboration d’un protocole d’utilisation de l’ACM. Il y a eu 4 cas chez lesquels la VA-ECMO par voie percutanée à l’aide de Cardiohelp (Getinge/Maquet, Wayne, New Jersey, É.-U.) a été utilisée ; 3 (75 %) ont survécu après l’hospitalisation de référence.

**Conclusions:**

Cette analyse a démontré la faisabilité de la mise en place d’une équipe de choc dans les régions éloignées du nord de l’Ontario, qui a permis d’utiliser efficacement la VA-ECMO dans un centre d’une circonscription hospitalière rurale non négligeable. Cette initiative aide à remédier à l’écart des résultats en soins cardiaques entre les régions rurales et urbaines de l’Ontario.

Cardiogenic shock (CS) mortality remains high despite the advancement in acute cardiac care, irrespective of the extent of immediate coronary revascularization.^1^ The outcomes are notably worse for patients in Northern Ontario than those in Southern Ontario, including 1-year mortality after myocardial infarction (MI).^2^ Besides several geographic and logistical challenges, the standard of care for patients presenting with ST-segment myocardial infarction (STEMI) differs between the 2 regions. Although patients presenting with STEMI in Southern Ontario undergo primary percutaneous coronary intervention (PPCI), a significant proportion of patients presenting with STEMI in Northern Ontario are still treated with thrombolytic therapy because of the limited access to emergency cardiac catheterization. Compared with PPCI, thrombolytic therapy is a less effective reperfusion strategy, with higher odds of death and reinfarction. As such, hospitals that care for patients with post-thrombolysis STEMI must have robust pathways in place for more prompt recognition and effective management of CS.

## Background

Health Sciences North (HSN) is a major referral centre that serves patients across Northeastern Ontario, a large geographic area, which is rural and medically underserved. Historically, patients at HSN who developed CS and required mechanical circulatory support (MCS) were managed with intra-aortic balloon pumps (IABPs). Although the use of IABPs in CS has been widely practiced, evidence for its efficacy in this setting is lacking.^3^ As such, some hospitals have moved to using Impella microaxial catheter-mounted pumps (Abiomed, Danvers, MA) to support some patients in CS. The combination of promising initial outcome data with its ease of use has led to the popularity of Impella rocketing, particularly in the United States. However, more recent data suggest that its use is associated with higher rates of adverse outcomes and elevated costs.^4^ Devices that assist the left ventricle (LV), such as IABP and Impella, may augment cardiac output. However, they fail to address other deleterious processes associated with CS, such as right ventricular (RV) dysfunction or profound hypoxemia. Veno-arterial extracorporeal membrane oxygenation (VA-ECMO) allows control of gas exchange, body temperature, and hemodynamic support. In addition, the use of VA-ECMO was associated with a significant survival benefit among patients with cardiac arrest and CS.^5^

## Methods

The objectives of this analysis included a review of the institutional use of Impella in the management of CS and related outcomes, excluding those who required MCS to assist weaning from cardiopulmonary bypass or as an adjunct during planned high-risk PCI. In addition, the analysis aimed to explore the feasibility of establishing a cardiogenic shock team (CST) to guide MCS decisions and to help establish VA-ECMO as an adjunctive tool in managing CS.

The primary outcome was in-hospital mortality. Secondary outcomes included MCS-related ischemic and hemorrhagic complications as well as the rates of successful MCS weaning. The CST was composed of specialists, including interventional cardiologists, cardiac surgeons, critical care physicians, perfusionists, and anaesthetists. These specialists commonly manage cardiogenic shock requiring MCS at the institution*.* Following consultation with several Canadian centres with expertise in MCS, the consensus of the CST was to develop a protocol to enable the use of VA-ECMO. Shock-team activation was typically initiated by phone conversations among various shock-team members but later evolved to using a secured text message group. The team decided on management, including candidacy for MCS, and further opinion from the regional transplant centres was sought in case of controversy. MCS cannulation was planned in the catheterization laboratory with the availability of an interventional cardiologist, cardiac surgeon, and perfusionist. Patients were admitted to the cardiovascular intensive care unit afterward for further care. Upon clinical and hemodynamic recovery, pharmacologic pressors were weaned first, followed by a gradual lowering of ECMO flow rates by 0.5 to 1 L/min, dependent on the patient’s condition until a rate of 2.5 L/min with monitoring of biventricular function using transesophageal echocardiography. In addition, the ventilatory requirement was also monitored. Inotropic agents were added as necessary to facilitate VA-ECMO weaning and decannulation. The institutional research ethics board approved the study.

## Results

We identified 15 patients with CS who received Impella between 2015 and 2021 (Table 1). The average age was 65 years (standard deviation [SD]: 13), and 8 patients (53%) were female. CS was ischemic in 12 patients (80%). For those with STEMI (8 patients), the median time from first medical encounter to catheterization laboratory arrival was 24 hours (9 to 26 hours); 2 had evidence of ventricular septal rupture. The median baseline lactate level was 5 mmol/L (3-8 mmol/L). All Impella devices were percutaneously inserted via the femoral artery approach. Impella CP (cardiac power) was used in all but 1 patient who received Impella 2.5 (Table 1). Thirteen patients (87%) died during the index hospital stay post-Impella because of progressive circulatory failure, with 3 patients (21%) suffering from vascular access compilations (Table 1).

**Table 1:** Clinical details of patients with CS who received Impella∗ and VA-ECMO

Age/Sex∗	Clinical course	Cardiac arrest pre- MCS/SCAI stage	RV-D	In-hospital death (cause of death)	Other remarks	Retrospective shock-team recommendations on MCS decisions
Impella						
40M	STEMI post-thrombolytics, recurrent ventricular fibrillation, CS. Femoral access for angiography with no PCI performed. Impella CP was inserted. Onset of symptoms to catheterization laboratory arrival was 9 hours. Baseline lactate 5 mmol/L.	Yes SCAI stage C	No	Yes (progressive shock)	Concerns regarding flow and position	VA-ECMO instead of Impella to facilitate PCI, especially given recurrent ventricular arrhythmia
65M	Possible myocarditis, with no significant coronary artery disease. Femoral access for angiography followed by insertion of Impella CP. Baseline lactate 15 mmol/L.	Yes SCAI stage C	Yes	Yes (progressive shock)	–	VA ECMO given RV dysfunction
60F	NSTEMI , iatrogenic left main dissection during PCI resulting in CS followed by insertion of Impella CP. PCI was performed via radial approach. Baseline lactate was not available.	Yes SCAI stage D	No	Yes (progressive shock)	Concerns regarding flow and position	VA-ECMO for CS in cases of catastrophic PCI complications
70F	Late anterior STEMI with occluded LAD with VSD, Impella CP was inserted. Femoral access for angiography. No thrombolytic was given. Onset of symptoms to catheterization laboratory arrival was ∼24 hours. Baseline lactate was not available.	No SCAI stage C	Yes	Yes (progressive shock)	-	Pharmacologic support, given late presentation, lack of revascularization, and RV dysfunction
35M	Anterior STEMI, thrombolysis followed by PCI to LAD. Impella was inserted and removed shortly thereafter after because of vascular complication. Further deterioration given pulmonary hemorrhage requiring VV-ECMO, which was decannulated later. PCI was performed via femoral access. Onset of symptoms to catheterization laboratory arrival was ∼4 hours. Baseline lactate of 5	Yes SCAI stage D	No	Survived	Large right femoral artery pseudo-aneurysm	Medical management without MCS; Impella was a reasonable option
55M	Anterior STEMI caused by stent thrombosis with CS managed with thrombolysis followed by rescue PCI followed by insertion of Impella CP. PCI was performed via femoral approach. First medical encounter to catheterization laboratory arrival was ∼48 hours. Baseline lactate 1 mmol/L.	Yes SCAI stage C	No	Yes (progressive shock)	–	Impella was a reasonable initial choice, with upgrade to VA-ECMO upon deterioration
65M	Severe chronic ischemic cardiomyopathy with acute decompensation. Femoral access for angiography. Impella CP was inserted. Baseline lactate of 8 mmol/L.	No SCAI stage C	Yes	Yes (progressive shock)	Issues with Impella flow	Medical management without MCS
75F	STEMI post-thrombolysis with VSD and CS followed by insertion of Impella 2.5, which was continued after VSD surgical repair. Femoral access for angiography. First medical contact to catheterization laboratory arrival was ∼26 hours. Baseline lactate of 8 mmol/L.	No SCAI stage D	Yes	Yes (progressive shock)	Issue with Impella position	IABP, as Impella is contraindicated with VSD
75F	STEMI post-thrombolysis with CS underwent PCI followed by IABP and Impella CP. Femoral access for angiography. First medical contact to catheterization laboratory arrival was ∼10 hours. Baseline lactate of 7 mmol/L.	No SCAI stage C	No	Yes (progressive shock)	–	MCS decision was reasonable
75F	STEMI presenting with VSD, and CS, followed by insertion of Impella CP. Patient's condition continued to deteriorate before surgical repair. First medical encounter to catheterization laboratory arrival was ∼24 hours. Baseline lactate 4 of mmol/L.	No SCAI stage D	No	Yes (progressive shock)	–	IABP, as Impella is contraindicated with VSD
60F	STEMI post-thrombolysis with ventricular fibrillation and CS underwent PCI followed by insertion of Impella CP. Femoral access for angiography. First medical encounter to catheterization laboratory arrival was ∼24 hours Lactate not available.	Yes SCAI stage C	No	Yes (progressive shock)	Issue with position of Impella	VA-ECMO, given recurrent ventricular arrhythmia
50M	Viral cardiomyopathy and rapid atrial fibrillation, severe LV dysfunction. Baseline lactate of 3 mmol/L.	No SCAI stage D	No	Yes (cerebellar hemorrhage)	–	VA-ECMO and early transfer to regional transplant centre for advanced heart-failure therapy
80F	Catheter-induced left main artery thrombosis or dissection resulting in CS underwent PCI followed by insertion of Impella CP. PCI was performed via femoral access. Baseline lactate was not available.	Yes SCAI stage E	No	Yes (circulatory failure and pulmonary hemorrhage)	Access-site bleeding	Supportive medical care with no MCS, given age and advanced stage of shock
70F	STEMI underwent PCI and insertion of Impella CP, which was weaned in 72 hours. PCI was performed via radial access. First medical encounter to catheterization laboratory arrival was ∼7 hours. Baseline lactate 3 mmol/L	Yes SCAI stage C	No	Survived	Massive transfusion related to groin-site bleeding	MCS decision here was reasonable
70M	NSTEMI with recurrent chest pain. Impella CP inserted for stabilization. Weaned from Impella in 48 hours, followed by recurrent shock. Femoral access for angiography. Baseline lactate 5 mmol/L.	Yes SCAI stage C	Yes	Yes Recurrent CS	–	Right heart catheterization to help guide initial MCS choice and subsequent weaning
VA-ECMO						
55F	Left main NSTEMI with CS with profound hypoxemia despite maximum pressors and ventilation. PCI to left main and VA-ECMO. Baseline lactate of 3 mmol/L.	Yes (at time of PCI) SCAI stage E		Survived	Accidental dislodgement of arterial cannula during CPR, requiring covered stent to right femoral artery and blood transfusion	–
65M	NSTEMI with biventricular failure and moderate-to-severe mitral regurgitation. Baseline lactate of 3 mmol/L.	No SCAI stage D	Yes	Survived		–
60M	NSTEMI, multivessel disease, no attempt at PCI, progressive shock, biventricular dysfunction, renal failure, liver dysfunction. Peripheral VA-ECMO insertion, transferred to local transplant centre. Baseline lactate of 4 mmol/L.	No SCAI stage D	Yes	Yes, hemorrhagic stroke after LVAD		–
40M	New nonischemic cardiomyopathy, progressive shock, Peripheral VA-ECMO insertion. Transferred to local transplant centre for further care. With recovery of LV function 8 days support. ICD inserted. Baseline lactate of 6 mmol/L.	Yes SCAI stage D	Yes	Survived, transferred to local transplant centre	Venous catheter dislodgement in catheterization laboratory, hematoma later infected. Arterial stenosis/mild claudication	–

IABP, intra-aortic balloon pump; CP, cardiac power; CPR, cardiopulmonary resuscitation; CS, cardiogenic shock; ICD, implantable cardioverter defibrillator; LAD, left anterior descending artery; LV, left ventricle; LVAD, left ventricular assist device; MCS, mechanical circulatory support; NSTEMI, non–ST-segment elevation myocardial infarction; PCI, percutaneous coronary intervention; RV-D, right ventricular dysfunction; SCAI, Society of Cardiovascular Angiography and Interventions; STEMI, ST-segment elevation myocardial infarction; VA-ECMO, venoarterial extracorporeal membrane oxygenation; VSD, ventricular septal defect; VV, veno-venous.

∗ Impella Systems (Abiomed, Danvers, MA).

**Figure 1:**
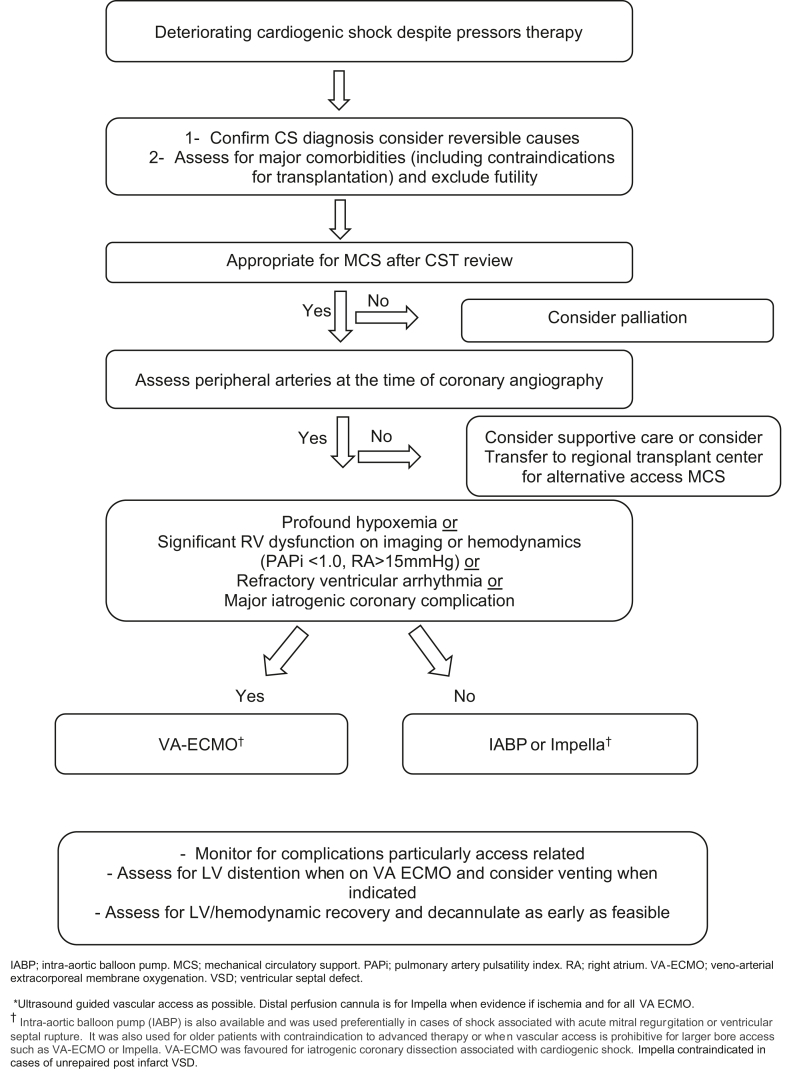
Mechanical circulatory support (MCS) decision-making protocol. CS, cardiogenic shock; CST, cardiogenic shock response team; IABP, intra-aortic balloon pump; LV, left ventricle; PAPi, pulmonary artery pulsatility index; RA, right atrium; VA-ECMO, veno-arterial extracorporeal membrane oxygenation; VSD, ventricular septal defect.

After consultation with several Canadian MCS centres, the CST team developed a local protocol to guide MCS use, particularly for VA-ECMO (Fig. 1). VA-ECMO was not offered to patients who suffered out-of-hospital cardiac arrest to increase the chances of successful use during the infancy of the CST. A "dry run" of VA-ECMO was carried out semielectively to build familiarity with the system and troubleshoot potential issues in a more controlled setting. An elderly patient with profound LV systolic dysfunction undergoing transcatheter aortic valve implantation was cannulated, and the circuit was set up to provide MCS in the event of acute deterioration. Following this case, the CST was prepared to use VA-ECMO emergently. Initially, a decision was taken only to provide VA-ECMO within routine working hours to ensure that support from other colleagues was available, with a plan to have 24-hour, 7-day coverage depending on resource availability.

There have been 4 cases in which percutaneous VA-ECMO using Cardiohelp has been used at HSN, with one patient (25%) suffering in-hospital mortality (Table 1). In 2019, the first VA-ECMO candidate, a 50-year-old female patient, presented to HSN in CS with profound hypoxemia and refractory ventricular fibrillation despite emergent left main artery PCI; therefore, CST decided on VA-ECMO. The patient’s LV function improved significantly and the patient was successfully decannulated 72 hours postpresentation. This patient is doing well and has been leading an active life for 4 years. Subsequent patients with CS who received VA-ECMO were shown in Table 1.

## Discussion

Although these cases demonstrated the benefit of a CST, several lessons were learned while setting up the team and the VA-ECMO service. We highlight a few that may be useful to other hospitals embarking on the same journey.

1. This is the first analysis, to our knowledge, that explores the feasibility of establishing a shock team in rural region such as Northern Ontario. The local protocol (Fig. 1) not only guides decision making for CS and MCS but also includes criteria for determining the appropriate disposition for MCS weaning, whether locally or through transfer to facilities offering advanced heart failure therapy.
2. Early recognition of patients at risk for CS and prompt transfer to a PCI-capable centre is critical. Ministry of Health and Long-Term Care (MOHLT) adopts the “Life or Limb” policy to indicate the urgency of patient transfer where the best effort is exerted to enable transfer within a 4-hour window. Although this is an important step, we believe this does not go far enough concerning high-risk patients with STEMI, particularly those with signs of thrombolysis failure and CS. We advocate for more resources to be made available to streamline and expedite the transfer of these critically ill patients.
3. In establishing a CST, communication with other centres with expertise in MCS was vital. Moreover, having a close working relationship with regional transplant centres was critical for decision making regarding advanced heart failure therapy, cardiac transplantation candidacy, or in cases of help for MCS weaning.
4. Awaiting robust evidence for use of the Impella system in CS, we believe that the suboptimal outcomes related to Impella here were multifactorial. Use of Impella in cases of significant RV dysfunction or for patients with postinfarct ventricular septal defect is contraindicated and likely contributed to the poor Impella outcomes. In addition, Impella size and postinsertion care are important determinants of outcomes that need to be considered.
5. Monitoring for ischemic and bleeding complications is prudent while on MCS. Accordingly, using radial access for PCI is vital, particularly given this population's higher rates of thrombolytic administration. In addition, attention to distal perfusion can further minimize MCS-related vascular complications. It is important to note that before establishing the shock team, none of the patients who received Impella had distal perfusion, which might have contributed to the poor outcomes associated with Impella. In our experience, switching from activated clotting time (ACT) to partial thromboplastin time (PTT) based anticoagulation significantly minimized bleeding and blood product transfusion while on VA-ECMO, without major ischemic complications.
6. It is essential to assess and monitor the development of LV distension carefully, as VA-ECMO increases afterload and LV wall tension. This can pose challenges for weaning off inotropic agents, and options to mitigate this include concomitant use of an IABP, Impella, or venting the left atrium to allow LV unloading.
7. Cardiac programs (and industry, when applicable) must monitor the local CS outcomes, including those related to various MCS devices. This helps guide appropriate patient selection, address issues related to device handling, and assist with staff education.

### Limitations

This analysis has several limitations, including being a single-centre experience with a relatively small sample size. In addition, the RV function assessment was mainly based on imaging. However, pulmonary artery catheter use was later incorporated as part of the protocol. We must point out that before the shock-team development, there was no standardization of care, including the availability of pre-MCS baseline lactate level. Moreover, the reported Impella experience here had predated the shock team's establishment, which may have had negative effect on the Impella experience. It is important to note that this is not a comparison between Impella and VA-ECMO but rather a description of positive outcomes associated with restructuring and standardizing the care received by the patient with CS, which we believe establishing the shock team helped to achieve.

## Conclusions

We demonstrated the feasibility of developing a CST that enabled the local use of VA-ECMO with good initial success. In addition, CST facilitated delivering appropriate, effective, and timely MCS in a nontransplant centre with a sizeable rural catchment area, using multidisciplinary teamwork and mentorship from a local cardiac transplant centre. This initiative can help bridge the existing gap in cardiac care and outcomes between patients residing in rural vs more urban regions of Ontario.
